# Report of Bureau of Organization, Etc. American Institute of Homœopathy

**Published:** 1891-01

**Authors:** 


REPORT OF THE BUREAU OF ORGANIZATION, REGISTRATION, AND STATISTICS OF THE AMERICAN INSTITUTE OF HOMŒOPATHY. Session of 1890. By Thos. Franklin Smith, M.D., 264 Lenox Avenue, New York.

This pamphlet of forty pages contains a complete list of all the homœopathic societies, dispensaries, hospitals, and colleges in the United States. It is valuable, therefore, to compilers of statistics and history of Homœopathy.

